# *Yersinia enterocolitica* in sheep - a high frequency of biotype 1A

**DOI:** 10.1186/1751-0147-54-39

**Published:** 2012-06-29

**Authors:** Karin Söderqvist, Sofia Boqvist, Georges Wauters, Ivar Vågsholm, Susanne Thisted-Lambertz

**Affiliations:** 1 Department of Biomedical Sciences and Veterinary Public Health, Swedish University of Agricultural Sciences, Box 7028, , SE-750 07, Uppsala, Sweden; 2 Research and Development Department, National Food Agency, Box 622, SE-751 26, Uppsala, Sweden; 3 Université Catholique de Louvain, Microbiologie, UCL 5492 avenue Hippocrate 54, 1200, Brussels, Belgium; 4 Karin Söderqvist, Section of Bacteriology and Food safety, Department of Biomedical Sciences and Veterinary Public Health, Swedish University of Agricultural Sciences, Box 7028, SE-750 07, Uppsala, Sweden

**Keywords:** *Yersinia enterocolitica*, Sheep, Biotype 1A, Zoonosis, Tonsil, Faeces

## Abstract

**Background:**

Pigs are regarded as the main reservoir for human pathogenic *Yersinia enterocolitica*, which is dominated by bioserotype 4/O:3. Other animals, including sheep, have occasionally been reported as carriers of pathogenic strains of *Y. enterocolitica*. To our knowledge, this is the first study performed in the Nordic countries in which the presence of *Y. enterocolitica* in sheep is investigated.

**Methods:**

Tonsils and faecal samples collected from sheep slaughtered on the island Gotland (Sweden) from September 2010 through January 2011 were analysed for presence of *Y. enterocolitica*. In an attempt to maximize recovery, several cultural strategies were applied. Various non-selective media were used and different temperatures and durations of the enrichment were applied before subculturing on Cefsulodin Irgasan Novobiocin (CIN) agar. Presumptive *Y. enterocolitica* colonies were subjected to urease, API 20E and agglutination test. *Yersinia enterocolitica* isolates were biotyped, serotyped, and tested for pathogenicity using a TaqMan PCR directed towards the *ail*-gene that is associated with human pathogenic strains of *Y. enterocolitica.*

**Results:**

The samples collected from 99 *s*heep yielded 567 presumptive *Y. enterocolitica* colonies. Eighty urease positive isolates, from 35 sheep, were identified as *Y. enterocolitica* by API 20E. Thirty-four of 35 further subtyped *Y. enterocolitica* isolates, all from faecal samples, belonged to biotype 1A serotype O:5, O:6. O:13,7 and O:10. One strain was *Yersinia mollaretii* serotype O:62. No human pathogenic strains of *Y. enterocolitica* were found in the investigated sheep. Other species identified were *Y. kristensenii* (n = 4), *Y. frederiksenii/intermedia* (n = 3), *Providencia rettgeri* (n = 2), *Serratia marcescens* (n = 1) and *Raoultella ornithinolytica* (n = 1).

**Conclusions:**

This study does not support the hypothesis that sheep play an important role in transmission of the known human pathogenic *Y. enterocolitica* in the studied geographical region*.* However, because there are studies indicating that some strains of *Y. enterocolitica* biotype 1A may cause disease in humans, the relative importance of sheep as carriers of human pathogenic strains of *Y. enterocolitica* remains unclear. Tonsils do not appear to be favourable sites for *Y. enterocolitica* biotype 1A in sheep.

## Background

Human pathogenic strains of *Yersinia enterocolitica* cause yersiniosis, a food-borne zoonosis. It is a gastrointestinal pathogen causing symptoms which vary depending on the age of the host and the bioserotype of the infecting strain. The most commonly reported symptoms are diarrhea, vomiting, abdominal pain, and fever. There is also a considerable risk of sequelae; reactive arthritis and erythema nodosum are common [1,2] but inflammatory bowel disease and irritable bowel syndrome are also reported [3]. Yersiniosis is the third most commonly reported zoonosis in Sweden, as well as in the EU. Nearly all cases appear sporadically and outbreaks are very rare [4]. In Sweden, yersiniosis is notifiable, and from 2001 through 2010 the annual incidence for the whole country ranged from 3 to 9 cases per 100 000 inhabitants, but was 5 to 16 on the island Gotland [5]. It is important to note that approximately 30% of the cases reported in Sweden are children under five years of age [6].

Strains of *Y. enterocolitic*a traditionally associated with yersiniosis are classified into five biotypes: 1B, 2, 3, 4, and 5. Most of the reported yersiniosis cases in Europe are caused by *Y. enterocolitica* biotype 4, serotype O:3 [4]. Biotype 1A strains are widespread in the environment and are generally considered to be non-pathogenic [2]. There are, however, studies indicating that some of the 1A strains have the ability to cause disease in humans, although they lack the classical virulence markers [7,8].

There are various methods described for isolation of *Y. enterocolitica* but no single procedure exists that covers all common human pathogenic bioserotypes [9]. In sample matrices of food, environment, and asymptomatic animal carriers (faeces, tonsils etc.) a large number of various non-target micro-organisms constitute the background flora. Unfortunately, the currently available enrichment and plating media for isolation of pathogenic strains of *Y. enterocolitica* are not selective enough to repress the background flora which increases the risk of false negative results. PCR can be used to indicate the presence of the pathogen in a sample before subculture on a solid media and/or to examine isolated colonies to reveal the presence of a potentially pathogenic strain. PCR assays that detect the group of bioserotypes associated with human disease are available [10].

Strains that belong to bioserotypes associated with human disease have frequently been isolated from tonsils and fecal samples of domestic pigs. A number of studies indicate prevalences of bioserotype 4/O:3 isolated from tonsils ranging from 38 to 67% [11-13], while corresponding prevalences in samples of pig faeces range from 8 to 13% [12].

At least one out of 4 cases of yersiniosis appears to originate from other sources than pigs [14] and there is a need to investigate alternative putative sources. Different food-producing animals have been examined as reservoirs for *Y. enterocolitica,* but data on the prevalence in sheep is limited. Human pathogenic strains have been isolated from sheep samples in a few studies from other countries [15,16] and a genotype relationship has been established between strains isolated from humans and sheep in Great Britain, indicating that sheep may be a potential reservoir of human pathogenic strains of *Y. enterocolitica*[17].

In the Island of Gotland (Sweden), there has been a persistent elevated incidence of human yersiniosis and a large population of sheep suggesting that sheep could be a possible source. Hence, the objective of this study was to examine sheep as potential carriers of human pathogenic strains of *Y. enterocolitica.*

## Methods

### Study design and sample collection

About 3000 sheep older than one year (personal communication Örjan Hansson, Gotlands Slagteri AB) were slaughtered at Gotlands Slagteri AB during the study period from September 2010 through January 2011. The establishment is submitted to official control by the National Food Agency in Sweden. To detect a prevalence of human pathogenic *Y. enterocolitica* of at least 3% with a 95% Confidence Interval (CI), the number of samples to be collected (n = 97) was calculated using the software program Win Episcope 2.0 [18]. The supervisors at the abattoir were instructed to select the sheep for sampling at random. From each sheep, the two tonsils were sampled separately using clean plastic gloves and a sterile scalpel. In addition, approximately 10 g of faeces was collected from the colon or rectum of the same sheep, using an aseptic procedure. The slaughter numbers were recorded to enable tracing of the origin, if necessary. All samples collected were stored at approximately 8°C and transported chilled to the laboratory, situated at the Swedish University of Agricultural Sciences (SLU, Uppsala). Most samples arrived at the laboratory the day after sampling. In most cases analysis started on the day of arrival. If samples arrived immediately before a weekend they were stored in a cool incubator at 4°C. When storage was needed more than three days, samples were stored until start of analysis in a freezer at −18°C.

In addition, pathogenic strains of *Y. enterocolitica* isolated from patients contracting yersiniosis during the study period were collected and stored at the regional hospital in Gotland (Visby lasarett) before being sent to SLU for further analysis.

### Sample preparation

#### Culture and identification of *Y. enterocolitica*

The most efficient methods to detect *Y. enterocolitica* from sheep faeces and tonsils are not known. Therefore, in this study several method variations were tested.

**Figure 1:**
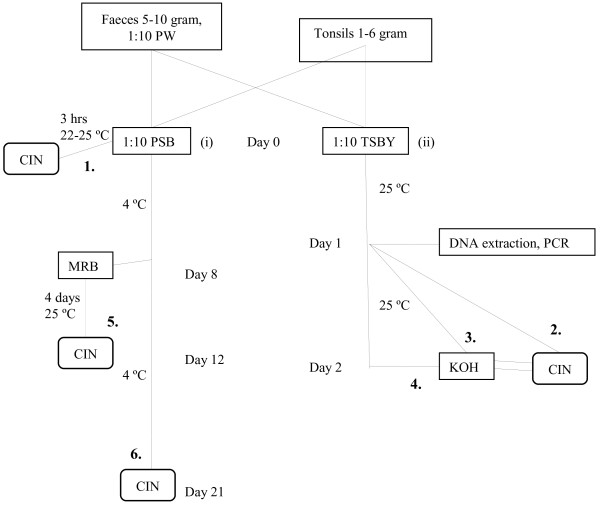
**Overview of the two culture methods used in this study, both somewhat modified from the original.** (i) A method based on the NMKL method no. 117 (1996), that includes cold enrichment, selective enrichment and subculture on a selective agar plate and (ii) a TaqMan-PCR based method (Thisted Lambertz, 2008) that includes enrichment overnight in 25°C, PCR analysis and subculture on a selective agar plate. The step indicated by dotted lines was performed if no characteristic pathogenic *Y. enterocolitica* colonies were visible on CIN agar day 1. Number 1–6 refer to the different subculturing (CIN) sampling steps as indicated in Table 1. CIN, Cefsulodin Irgasan Novobiocin; PW, Peptone water; PSB, Phosphate-buffered saline containing 2% sorbitol and 0.15% bile salts; TSBY, Tryptone-soya broth supplemented with yeast; KOH, Potassium hydroxide solution; MRB, Modified-Rappaport-broth.

At the laboratory the two tonsils from each sheep were pooled and cut into even smaller pieces with a sterile pair of scissors before being divided equally into two filter bags (Stomacker®lab system classic filter bag, http://www.Seward.co.uk). The tonsils were very small; about 0.5-3 g each. Before homogenised, the tissue in one of the bags was diluted 1:10 with PSB (Phosphate-buffered saline containing 2% sorbitol and 0.15% bile salts) and in the second diluted 1:10 with TSBY (Tryptone-soya broth supplemented with yeast). Samples were then processed further as outlined in Figure 1. Five to 10 g of a faecal sample, depending on the amount collected, was initially diluted 1:10 with Peptone water (PW) before being homogenised. After being equally divided in two filter bags, the portions of faecal homogenate were further diluted (1:10) with PSB and TSBY, respectively, and then processed as outlined in Figure 1.

The PSB homogenates were pre-incubated for 3 h at 22–25 °C before a 10-μl loop of the enrichment was subcultured on a Cefsulodin Irgasan Novobiocin (CIN) agar plate (Oxoid, CM 653 and SR 109), see pathway (i) in Figure 1. The PSB broth was incubated, this time at 4 ±1°C for 21 days (cold enrichment). After eight days, a subportion of 100 μl of the enriched culture was transferred into 10 ml of MRB (modified-Rappaport-broth). The mixture was homogenised and incubated at 25 ±1°C. After 4 days, 10 and 100 μl, respectively, of the MRB culture was subcultured on CIN-agar plates. When the cold enriched culture was 21 days old, 20 μl was subcultured on CIN-agar plates.

The TSBY homogenates were incubated at 25 ±1°C for 21 ±3 h, see pathway (ii) in Figure 1. To reduce the background flora, a 0.5-ml portion of the enrichment was transferred into 4.5 ml 0.5% potassium hydroxide solution (KOH) and mixed gently for 20 s before 10 μl of the mixture was subcultured on a CIN-agar plate. In parallel, 10 μl of the enrichment was subcultured directly onto CIN agar, i.e. without previous KOH treatment. If no characteristic pathogenic *Y. enterocolitica* colonies were visible on the CIN agar plate following KOH treatment after 24 h incubation of enrichment, a second plating on CIN agar following KOH treatment was performed after 48 h of enrichment.

All CIN-agar plates (1–6 in Figure ) were incubated at 30 °C for 21 ±3 h. Colonies with a deep red center (bull’s eye) surrounded by an outer transparent zone were considered presumptive *Y. enterocolitica.* Bull´s eye colonies were subcultured on segments of a non-selective agar plate to enable purity and were then transferred into tubes containing Urea-indole medium and incubated at 30 ±1°C for 24 ±3 h. Urease-positive cultures (pink colouring of the medium) were biochemically analysed using API 20E strips (bioMérieux, France) that were incubated at 30 °C for 24 h. Isolates identified as *Y. enterocolitica* were further tested with antisera against *Y. enterocolitica* O:3 and O:9 (Reagensia AB, Sweden). A selection of API 20E-identified *Y. enterocolitica* isolates were sent to the Microbiology laboratory, Université Catholique de Louvain, Brussels (Belgium) for biotyping according to Wauters et al. [19]. Serotyping was based on the O-antigens described in the antigenic scheme including 76 O-factors characterized among *Y. enterocolitica* and related species [20].

The collected human strains were biotyped with the following tests conducted: lipase (tween-esterase), acid production from salicin, xylose and trehalose, esculin hydrolysis, Voges-Proskauer and production of pyrazinamidase [19,21]. In addition, Congo Red-Brain Heart Infusion agarose plates (CR-BHO) were used to enable detection of the virulence plasmid [22].

All bioserotyped *Y. enterocolitica* strains collected in this study are stored in the NFA (The Swedish National Food Agency) Culture Collection.

### Bacterial reference strains

*Yersinia enterocolitica* 4/O:3, strain SLV-408 (CCUG 45643) was used as reference and positive control. This strain was originally isolated from frozen raw dog food, containing pig meat, and is commercially available.

#### DNA extraction and TaqMan PCR

In one of the analysis pathways (ii), a PCR analysis was included. Prior to PCR analysis DNA was extracted from bacteria cells. The BioROBOT EZ1 (Qiagen) system and associated kit (EZ1 DNA Tissue Kit (48), Cat. No. 953034) was used for the enriched culture and the Insta Gene Matrix (Bio-rad, 732–6030) fluid was used regarding lysis of the pure bacterial colonies identified by API 20E as *Y. enterocolitica*. Both extraction methods were performed according to the manufacturer’s instructions. A test portion of 5 μl of the DNA extraction alternatively lysed bacteria was used for PCR analysis. A TaqMan probe-based PCR method targeting the *ail*-gene [10] was used. In the PCR analysis, the reference strain SLV-408 was used as positive amplification control and sterile distilled water as negative control.

A pre-study was carried out to check the possible influence of PCR-inhibiting substances present in the matrices tonsil tissue and faeces, and to estimate an approximate detection level. The two sample matrices were inoculated with 10-μl portions of appropriate concentrations of an overnight (30 °C) BHI-broth culture of the reference strain, SLV-408, containing approximately 10^9^ cfu/ml, serial diluted ten-fold in PW to achieve levels of *Y. enterocolitica* ranging from 10^0^-10^4^ cfu/ml. Absence of pathogenic *Y. enterocolitica* in the matrices was confirmed by including a negative control sample in each test. The 1:10 diluted TSBY-inoculated samples were homogenised and incubated overnight at 25 ±1°C. DNA was extracted and PCR analysis performed. For each matrix, this test was run in duplicate. To estimate the number of *Y. enterocolitica* bacteria contained in the inoculums, 100-μl volumes (x3) from the dilutions 10^-6^ to 10^-8^ were subcultured on a non-selective agar medium and incubated overnight at 30 ±1°C, after which the colonies were counted.

## Results

### Culture and identification of *Y. enterocolitica*

Although tonsil and faecal samples were collected from 105 sheep after slaughter, only samples from 99 sheep were used. A group of samples from six sheep was delayed more than 48 h and therefore excluded from the study. In all, 567 colonies grown on CIN agar were identified as presumptive *Y. enterocolitica* and of those, 95 (17%) were urease positive. API 20E identified the majority (80/95) of the urease isolates as belonging to the species *Y. enterocolitica*, however, in varying degree according to the API species definition: four with ‘Very good identification’, 66 with ‘Good identification’; five with ‘Very good identification to the genus’ and five with ‘Doubtful profile’ although *Y. enterocolitica* was mentioned as one significant taxa.

The isolates identified as *Y. enterocolitica* (n = 80) all originated from faecal samples collected from 35 of the sheep (35%). One of these sheep also carried API 20E-identified *Y. enterocolitica* in the tonsils. Furthermore, four isolates were identified as *Y. kristensenii*, three as *Y. frederiksenii/intermedia*, two as *Providencia rettgeri*, one as *Serratia marcescens* and one as *Raoultella ornithinolytica*. None of the isolates became positive in agglutination tests with antisera O:3 or O:9. The first 35 out of the 80 API 20E-identified *Y. enterocolitica* isolates were bioserotyped. Out of the 35 isolates, 27 belonged to 1A/O:5, four to 1A/O:6, two to 1A/O:13,7 and one to 1A/O:10. One isolate was identified as *Yersinia mollaretii*, serotype O:62.

The number of *Y. enterocolitica* 1A strains recovered in all 6 subculturing (CIN) steps is given in Table 1. Culturing after enrichment in TSBY for one or two days combined with KOH treatment (no 3 and 4) were the most effective methods to isolate *Y. enterocolitica* 1A. Selective enrichment in MRB (no 5) was also effective. No *Y. enterocolitica* 1A was isolated after direct culture on CIN agar plates from pre-incubated PSB.

**Table 1:** Outcome of different subculturing steps

		**Number of strains**		
**Subculturing steps**	**Days of incubation**	**API 20E identified Ye**	**BT1A**	
1. PSB (i)	0 (3h)	3	0
2. TSBY (ii)	1	7	4
3. TSBY+KOH-treatment (ii)	1	17	8
4. TSBY+KOH-treatment (ii)	2	13	9
5. PSB+MRB (i)	8+4	18	8
6. PSB (i)	21	22	5
Total		80	34

The number of isolates identified as *Yersinia enterocolitica* (Ye) by API 20E and biotyped as 1A (BT1A) found at different subculturing (CIN) steps during the culture procedures applied.

(i), (ii) refers to the different pathways, shown in Figure 1. PSB, Phosphate-buffered saline containing 2% sorbitol and 0.15% bile salts; TSBY, Tryptone-soya broth supplemented with yeast; KOH, Potassium hydroxide solution; MRB, Modified-Rappaport-broth.

The human *Y. enterocolitica* isolates (n = 2) were trehalose- and Voges-Proskauer positive but lipase-, salicin-, xylose-, esculin- and pyrazinamidase negative; consequently they belonged to biotype 4. The strains were serotyped as O:3, according to the slide agglutination test. Both strains carried the virulence plasmid (CR-BHO).

### TaqMan PCR

The results of the prestudy showed that faecal matrix caused considerable PCR inhibition compared to tonsils. When using the EZ1 BioROBOT to extract DNA, acceptable levels of PCR detection for the two matrices were obtained after an initial dilution of the faecal samples 1:10. An extraction-elusion volume of 200 μl for the faecal samples and 50 μl for the tonsil samples were applied. Using this DNA extraction setup it was possible to detect approximately 10^2^ to 10^3^ cfu *Y. enterocolitica* per gram of faecal or tonsil sample.

The *ail*-gene was not found in any of the sample enrichments, and was not detected when testing the urease-positive *Y. enterocolitica* isolates (n = 95). However, in the two human strains collected in Gotland, the *ail*-gene was detected.

## Discussion

This study shows the presence of *Y. enterocolitica* in tonsil and faecal samples of sheep. The sheep were bred and slaughtered on Gotland, a Swedish island that also has swine and cattle production. In this study no human pathogenic biotype was isolated and no *ail*-gene was detected. However, a whole set of *Y. enterocolitica* biotype 1A was found, where 34 out of 35 bioserotyped isolates belonged to this biotype. The absence of human pathogenic *Y. enterocolitica* strains in this study is at variance with findings in studies outside Sweden. Bioserotype 4/O:3 has been isolated from the rectal content in 12% of lambs (33/281) in a survey from New Zealand [15] and bioserotype 3/O:5,27 has been isolated from faeces in 3% of sheep (30/973) in a British study [23]. A high seroprevalence (56%) of yersinia antibodies was found in sheep in Northern Germany [24]. However, another German study detected the *ail*-gene only in 5% (3/64) of the sheep tonsil samples and in none of 200 analysed faecal samples [12]. A study in Nigeria detected the *ail*-gene in 1% (2/200) of faeces samples in investigated sheep [25].

The incidence of yersiniosis was low during the investigation period (mainly 2010), both in Gotland and in Sweden as a whole. Still, the incidence in Gotland was higher than the Swedish average [5]. There were two reported cases of human yersiniosis in the study area during the study period, and bioserotype 4/O:3 was isolated from both of them. During 2011, *Y. enterocolitica* 2/O:9, carrying the *ail*-gene, was isolated from the faecal samples of 4 sheep from Skåne in southern Sweden (personal communication, Elisabeth Bagge, National Veterinary Institute). During the same year there were 11 reported human cases of yersiniosis caused by *Y. enterocolitica* 2/O:9 in Sweden (personal communication Margareta Löfdahl, SMI).

Strains of biotype 1A are ubiquitous in the environment and generally considered to be non-pathogenic [2]. The latter is, however, subject to discussion [8]. Some studies suggest that biotype 1A lacks clinical significance, because biotype 1A has been isolated more frequently in healthy subjects than in patients with intestinal disease [7]. Other studies suggest the opposite; that *Y. enterocolitica* biotype 1A *is* associated with clinical disease [8,26,27]. In recent studies from Switzerland and Finland, the prevalence of isolated *Y. enterocolitica* biotype 1A from yersiniosis-patients was high, 40 and 65% respectively. The biotype 1A patients were older and had another spectrum of symptoms than patients from whom traditionally pathogenic biotypes of *Y. enterocolitica* were isolated [8,28]. A few biotype 1A strains were also found amongst the reported cases from 2008 to 2011 in Sweden (Margareta Löfdahl, SMI, personal communication). Cold enrichment is not used for clinical samples in Sweden which could be one reason for the less frequent finding of biotype 1A strains in Sweden compared to in Finland where they often use this method [28].

One of the classic virulence factors, the *ail*-gene, has been detected in low frequencies in strains of biotype 1A [29,30]. Kraushaar et al. [31] found that the *ail* region of a biotype 1A strain differed from the corresponding region of pathogenic strains. Furthermore, there are indications that biotype 1A strains of clinical origin from human cases have characteristics that differ significantly from those that are not from human cases [7]. Batzilla et al. [32] found genes in biotype 1A encoding known and suspected virulence-associated determinants, indicating their opportunity to establish infection in immunosuppressed patients. The role of *Y. enterocolitica* biotype 1A in causing disease in humans is debated and a priority for research is to investigate virulence mechanisms other than those currently known.

It was not obvious which bioserotypes of *Y. enterocolitica* the samples in this study would harbour. Therefore isolation methods were chosen that allowed a broad spectrum of bioserotypes to grow. Mainly non-selective culture media were used in this study (TSBY and PSB). In parallel, a selective enrichment medium was also used (MRB). The latter is known to favour growth of the human pathogenic serotypes O:3 and O:9 and inhibits most of the background flora [33]. However, in the present study the use of MRB did not seem to inhibit other *yersiniae*. In fact, MRB enrichment was effective in culturing biotype 1A (see Table 1). CIN agar is the best available choice of solid medium for isolation of different bioserotypes of *Y. enterocolitica*[33]. Unfortunately, several other bacteria also grow on the CIN medium, and the selection of presumptive *Y. enterocolitica* is difficult. In the present study there was a reduction of the presumptive colonies from 567 to 95 after urease testing, indicating the difficulties in selecting colonies with typical morphology. It appears that when studying new potential reservoirs for *Y. enterocolitica* it is important to use methods that do not discriminate certain serotypes and bias the results. In the British study, cold enrichment in PSB broth was the only method used and KOH-treatment was not used to reduce the background flora. Biotype 1A was the most common strain isolated, 6.6% of the sheep carried this biotype [23]. Our result indicates a higher prevalence of biotype 1A than observed in the British study.

In the present study, traditional culturing methods were combined with real-time PCR detecting the *ail-*gene. A BioROBOT EZ1 was used to extract DNA prior to PCR, which is more effective than extraction with the DNeasy Blood and Tissue kit (Qiagen GmbH, Hilden Germany). The BioROBOT EZ1 only requires 200 μl of the sample, while the DNeasy Blood and Tissue kit requires 1 ml to yield the same PCR result, i.e. the same Ct value when testing low value of *Y. enterocolitica* bacteria (approx.10^2^ cfu/ml). The selective and non-selective isolation methods used, combined with BioRobot extraction and TaqMan PCR, optimizes the chance to find *Y. enterocolitica.*

API 20E is not optimally developed for identification of the non-pathogenic strains of *Y. enterocolitica*. A correct identification of the non-pathogenic strains therefore requires additional tests [16], as demonstrated by the results obtained in this study where some of the isolates that had doubtful profiles were later identified as *Y. enterocolitica* biotype 1A. One isolate that was classified as *Y. enterocolitica* in API 20E with ‘very good identification’ was in fact *Yersinia mollaretii* which is phenotypically closely related to *Y. enterocolitica. Yersinia mollaretii* was earlier described as *Y. enterocolitica* biotype 3A [34].

## Conclusions

Sheep from Gotland do not appear to be important in the transmission of traditionally pathogenic strains of *Y. enterocolitica* to humans. There is a high frequency of *Y. enterocolitica* biotype 1A in the faecal samples from the investigated sheep but not in the tonsils. It appears that when studying new potential reservoirs for *Y. enterocolitica* it is important to use methods that do not discriminate certain serotypes and therefore bias the results. The zoonotic potential of biotype 1A has received more attention recently and identification of pathogenic subgroups is a future challenge for research.

## Competing interests

The authors declare that they have no competing interests.

## Authors’ contributions

SB, STL and IV initiated and designed the study, STL being responsible for the bacteriological analysis. KS carried out the bacteriological analysis, and drafted the manuscript. GW gave assistance on the bioserotyping. All authors were involved in the interpretation of results and drawing of conclusions, and have given helpful advice in writing the paper. All authors have read and approved the final manuscript.
